# Does Clinical and Biochemical Thyroid Dysfunction Impact on Endometrial Cancer Survival Outcomes? A Prospective Database Study

**DOI:** 10.3390/cancers13215444

**Published:** 2021-10-29

**Authors:** Chloe E. Barr, Kelechi Njoku, Leo Hotchkies, Neil A. J. Ryan, Y. Louise Wan, David A. Davies, Salman Razvi, Emma J. Crosbie

**Affiliations:** 1 Department of Obstetrics and Gynaecology, Manchester University NHS Foundation Trust, Manchester Academic Health Science Centre, Manchester M13 9WL, UK; Chloe.Barr@mft.nhs.uk; 2 Division of Cancer Sciences, Faculty of Biology, Medicine and Health, University of Manchester, Manchester M13 9WL, UK; kelechi.njoku@manchester.ac.uk (K.N.); leo.hotchkies@manchester.ac.uk (L.H.); neil.ryan@manchester.ac.uk (N.A.J.R.); Louise.Wan@mft.nhs.uk (Y.L.W.); 3 Department of Laboratory Medicine, Manchester University NHS Foundation Trust, Manchester M13 9WL, UK; David.Davies@mft.nhs.uk; 4 Translational and Clinical Research Institute, Newcastle University, Newcastle upon Tyne NE1 7RU, UK; salman.razvi@newcastle.ac.uk

**Keywords:** biomarker, endometrial cancer, hypothyroidism, prognosis, recurrence, survival

## Abstract

**Simple Summary:**

Endometrial cancer is the most common gynaecological cancer in high-income countries. Most women are diagnosed early and have an excellent prognosis, but those with advanced or recurrent disease have poor outcomes. The aim of this study was to determine whether clinical or biochemical thyroid dysfunction may contribute to survival outcomes following diagnosis and treatment for endometrial cancer. We analysed clinical data and serum thyroid hormone status of 333 women treated for endometrial cancer at a specialist cancer centre and followed up for a median of 35 months. Women with a diagnosis of hypothyroidism had improved overall, cancer-specific, and recurrence-free survival compared to those without. This may have important implications for our understanding of the mechanisms underpinning biologically aggressive disease and offer opportunities for therapeutic intervention.

**Abstract:**

Endometrial cancer is the commonest gynaecological malignancy in developed countries, and women presenting with high risk or advanced disease have poor outcomes. Thyroid hormones play a key role in cellular metabolism and can influence cancer growth and invasion. Our aim was to evaluate the association between clinical and biochemical thyroid dysfunction and endometrial cancer survival outcomes. This was a prospective cohort study of women treated for endometrial cancer at a specialist centre. Clinical diagnosis of hypothyroidism was based on clinical and biochemical assessment, verified by general practitioner (GP) records. Pre-treatment serum samples were tested for thyrotropin (TSH), thyroid hormones (free T4 and total T3), and thyroid peroxidase antibodies. Kaplan–Meier survival estimates and log-rank tests were used to compare survival between groups, while Cox regression was used for multivariable analysis, adjusting for known confounders and effect modifications. In total, 333 women with median age and body mass index (BMI) of 66 years (interquartile range (IQR) 56, 73) and 33 kg/m^2^ (IQR 27, 41) respectively were included. A total of 51 (15.3%) women had a diagnosis of hypothyroidism, 39 (11.9%) had biochemical evidence of overt or subclinical hypothyroidism. Median follow-up was 35 months (IQR 21, 45) with 38 (11.7%) relapses and 50 (15.0%) deaths. Women with a diagnosis of hypothyroidism had improved overall survival (adjusted HR = 0.22, 95%CI 0.06–0.74, *p* = 0.02), cancer-specific survival (adjusted HR = 0.21, 95%CI 0.05–0.98, *p* = 0.04) and fewer recurrences (adjusted HR = 0.17, 95%CI 0.04–0.77, *p* = 0.02) than those who did not. Confirmatory studies should explore underlying mechanisms and the potential for therapeutic exploitation.

## 1. Introduction

Endometrial cancer is the most common gynaecological malignancy in the United Kingdom (U.K.). Since the early 1990s, the incidence of endometrial cancer in the U.K. has risen by 55%, and deaths from the disease by 23%, despite improvements in overall survival [1]. Endometrial cancer has a generally good prognosis because three-quarters of women are diagnosed with early-stage, curable disease [2]. However, a significant minority of women present with advanced-stage disease when outcomes are extremely poor. There is an urgent need to better understand factors that influence survival to reduce the impact of the rising burden of endometrial cancer [3].

Thyroid hormones play a key regulatory role in many physiological processes, including cellular growth, differentiation, and metabolism [4]. Thyroxine (T4) stimulates tumour growth in several pre-clinical and animal models, an effect reversed by pharmacological or surgical thyroid hormone suppression [5,6,7]. Drug-induced hypothyroidism has been shown to improve survival during the treatment of renal cell carcinoma [8,9,10]. Accordingly, epidemiological data indicate that hypothyroidism is associated with improved survival outcomes in cancers of the breast [11,12], lung [13], and ovary [14], as well as metastatic brain disease, irrespective of the site of the primary tumour [15]. Conversely, hyperthyroid states are associated with poor prognosis from tumours of multiple types [16,17,18,19].

Despite an extensive body of work in other tumour sites, few studies have looked at the association between thyroid dysfunction and survival outcomes in endometrial cancer. The generally good prognosis means that large cohorts and prolonged follow-up are required to demonstrate any effect. Seebacher et al. found that baseline TSH > 2.5 mU/L was independently associated with poor disease-specific survival in 199 endometrial cancer patients from two centres in Austria [20]. These data contradict the expected relationship between thyroid hormones and survival outcome based on studies of other tumour types, although the literature is mixed [21,22]. The Seebacher study was relatively small with just 26 women with TSH > 2.5 mU/L and failed to show the expected associations of advancing age and histological subtype on endometrial cancer survival. Given the well-documented association between hypothyroidism and female sex, as well as the three major risk factors for endometrial cancer, specifically age, obesity, and type 2 diabetes mellitus [23,24], the prevalence of diagnosed and undiagnosed hypothyroidism is expected to be high amongst women with endometrial cancer, and any relationship with survival outcomes is therefore worthy of further investigation, particularly given the potential therapeutic implications.

The aim of this study was to evaluate the impact of clinical and biochemical thyroid dysfunction on survival outcomes in a large cohort of women with endometrial cancer.

## 2. Materials and Methods

### 2.1. Study Population

Women treated for endometrial cancer at Manchester University NHS Foundation Trust (MFT) between 2013 and 2018, who had donated clinico-pathological data, a pre-treatment serum sample and provided written, informed consent for future research [25], were eligible for inclusion (North West Research Ethics Committee reference 15/NW/0733). A clinical diagnosis of hypothyroidism was based on clinical and biochemical assessment, conducted at their initial presentation prior to endometrial cancer diagnosis, verified by GP records, and further confirmed by treatment with levothyroxine. Primary treatment was surgical for most women (total hysterectomy and bilateral salpingo-oophorectomy), with adjuvant chemotherapy/radiotherapy in accordance with international guidance [26]. Primary progestin therapy was used to preserve fertility or because women were unfit for surgery, followed by hysterectomy as required. A few women underwent primary palliative radiotherapy. Baseline clinical data included age, body mass index (BMI, kg/m^2^), comorbidities, current medications, date of diagnosis of endometrial cancer, treatment modality, and receipt of adjuvant treatment, including vault brachytherapy, external beam radiotherapy, and chemotherapy. Pathology data related to the hysterectomy specimen or diagnostic sample, where primary treatment was non-surgical and included histological subtype, FIGO (2009) stage, grade, lymphovascular space invasion (LVSI), and depth of myometrial invasion. FIGO staging was based on imaging if primary treatment was non-surgical. Women underwent routine clinical follow-up at 3-monthly (first 3 years), 6-monthly (4th year), and 12-monthly intervals (final year). Local recurrence was treated by surgery or radiotherapy as appropriate and distant recurrence by systemic hormones or chemotherapy [26]. At 5 years, women who remained recurrence-free were discharged from routine follow-up. Survival data were collected from clinic letters, GPs, and death certificates, as appropriate. Overall survival (OS) was the time between diagnosis and death or last follow-up, with no restriction by cause of death. Cancer-specific survival was defined as the time from diagnosis until death from endometrial cancer or last follow-up, censored on the date of death from other causes. Recurrence-free survival was measured from the end of primary treatment to first recurrence, death, or last follow-up.

### 2.2. Laboratory Assays

Pre-treatment serum was collected, processed, and stored at −80 °C in the MFT Biobank until analysed. Serum TSH, free T4 (FT4), and total T3 (TT3) levels were measured using chemiluminescent enzyme immunoassays on the Fujirebio Lumipulse G600II analyser (Fujirebio Europe N.V., Gent, Belgium) in the MFT gynaecological oncology research laboratory. Serum thyroid peroxidase antibody levels were measured by ImmunoCAP thyroid peroxidase fluorescence enzyme immunoassay on the Phadia 250 analyser (Thermo Fisher Scientific, Waltham, MA, USA) in the MFT clinical immunology laboratory. The British Thyroid Association’s normal clinical reference ranges were TSH 0.4–4.5 mU/L, FT4 9.0–25.0 pmoL/L, and TT3 1.2–2.6 nmoL/L [27]. The upper limit of normal for thyroid peroxidase (TPO) antibody was 60 IU/mL as per the manufacturer’s instructions.

### 2.3. Statistical Analysis

Descriptive analyses were performed using the χ^2^ test and Kruskal–Wallis one-way analysis of variance as appropriate, with the normality of data distribution assessed using the Shapiro Wilk test. Survival was analysed according to baseline thyroid hormone levels, using TSH as a continuous variable and following categorisation into two groups based on a TSH cut-off of 4.5 mU/L, baseline history of hypothyroidism, and categorisation of baseline thyroid function tests into clinical thyroid status to enable clinically meaningful results. All study co-variables were treated as categorical variables except for age and BMI, which were also considered as continuous variables. Survival rates were calculated using Kaplan–Meier estimation, and the differences in survival between groups were assessed using the log-rank tests. Cox proportional hazards regression analysis was used to model the probability of survival by TSH prognostic categories while adjusting for confounding and effect modifications. The model was adjusted for prognostic variables known to be associated with poor endometrial cancer survival from the literature and included age, BMI, socioeconomic deprivation quintiles, history of type 2 diabetes mellitus, history of thyroxine replacement at baseline, endometrial cancer histological subtype, lymphovascular invasion (LVSI), depth of myometrial invasion, stage, grade and type of treatment received. Hazard ratios (HRs) with 95% confidence intervals (CI) were reported for both univariable and multivariable analyses. Interactions were tested within the regression framework, and the likelihood ratio test was used to assess for nesting effects. Confounding was assessed by changes in the hazard coefficients following the introduction of other covariates in the regression model. Statistical models were built in a step-wise fashion based on results of univariable analysis, prior knowledge of the literature, and findings from likelihood ratio tests with the ultimate goal of achieving model parsimony. The assumption of proportional hazards was assessed and met for all models. *p*-values of <0.05 were considered statistically significant. The statistical package STATA (StataCorp. 2019. Stata Statistical Software: Release 16. StataCorp LLC: College Station, TX, USA) was used for all analyses.

## 3. Results

### 3.1. Descriptive Characteristics of the Study Population

The study population comprised 333 women with a median age and BMI of 66 years (IQR 56, 73) and 33 kg/m^2^ (IQR 27, 41), respectively (Table 1). The modal social deprivation quintile was quintile 1 (most deprived), representing 37.8% (125/331) of the population [28]. Most women were overweight or obese (285/333, 85.6%) with associated comorbidities, including hypothyroidism (51/333, 15.3%) and type 2 diabetes mellitus (59/333, 17.7%). The majority had low-grade (239/333, 71.7%), early-stage (256/333, 76.9%) endometrial cancer of endometrioid histological subtype (268/333, 80.5%). Fewer women had high-grade histological subtypes (serous (7.2%), clear cell (3.6%), carcinosarcoma (6.3%)). Primary treatment was surgery in 80.5% (268/333) and hormone therapy for fertility sparing (25/333, 7.5%) and surgical fitness (37/333, 11.1%) reasons, respectively. Just over half (13/25) of those managed conservatively for fertility sparing reasons received a hysterectomy during the study period. Approximately 40% (132/333) had adjuvant radiotherapy and/or chemotherapy. Median follow-up was 35 months (IQR 21, 45), 38 (11.7%) women recurred, and 50 (15.0%) died, while the remainder were alive or censored as of 31 March 2020. Deaths were either from endometrial cancer (33/50, 66%) or other causes (17/50, 34%).

### 3.2. Clinical and Biochemical Thyroid Dysfunction at Baseline

Clinical and biochemical patterns based on baseline thyroid function tests were categorised according to the British Thyroid Association guidelines (Table 2). Most women were euthyroid at diagnosis, including 88% (240/282) of those with no prior history of hypothyroidism and 58% (28/51) of those with a clinical history of hypothyroidism. Women with a history of hypothyroidism were more obese (median BMI 36 versus 32, *p* = 0.013) than those without. There was no statistically significant association between history of hypothyroidism and age (*p* = 0.411), social deprivation quintile (*p* = 0.274), history of diabetes mellitus (*p* = 0.743), histological subtype of endometrial cancer (*p* = 0.469), FIGO stage (*p* = 0.318), LVSI (*p* = 0.301), depth of myometrial invasion (*p* = 0.864), or tumour grade (*p* = 0.284).

The most common biochemical thyroid abnormality was subclinical hypothyroidism, seen in 27% (13/51) of women with a history of hypothyroidism (all of whom were on exogenous thyroxine treatment) and 8.5% (23/282) of women with no history of hypothyroidism. Baseline TSH levels ranged from 0.003 to 40.0 mU/L with a median TSH of 2.1 mU/L and IQR of 1.40–3.11 mU/L. TPO antibodies were positive in 35 (10.5%), negative in 286 (85.9%) and not available for 12 (3.6%) women. Approximately 30% (15/49) of women with a clinical history of hypothyroidism had positive TPO antibodies at baseline compared to 7% (20/272) of those with no prior history of thyroid dysfunction (*p* < 0.001). About 12% (39/329) of women had TSH levels >4.5 mU/L. There was no evidence of an association between baseline TSH > 4.5 mU/L and age (correlation coefficient = 0.01, *p* = 0.82), BMI (correlation coefficient = 0.06, *p* = 0.30), obesity status (*p* = 0.940), social deprivation quintile (*p* = 0.823), history of diabetes mellitus (*p* = 0.262), type of endometrial cancer (*p* = 0.566), FIGO stage (*p* = 0.939), LVSI (*p* = 0.931), depth of myometrial invasion (*p* = 0.971), or grade of endometrial cancer (*p* = 0.438).

The ft4 (pmoL/L)/T3 (nmoL/L) ratio was computed to assess the adequacy of intracellular conversion of free T4 to active T3 and its potential effect on survival outcomes. Ft4/T3 values ranged from 3.9 to 20.4 with a median of 8.2 (IQR 7.1, 10.4). There was a statistically significant difference in the ft4/T3 values between women with a history of hypothyroidism compared to those without (median 10.7 (IQR 7.8, 12.6) vs. median 8.1 (IQR 7.0, 9.6) respectively, *p* < 0.001).

### 3.3. Overall Survival Outcomes

Crude overall survival estimates and unadjusted hazard ratios based on univariable analyses are summarised in Table 3. Overall survival for the study cohort was 96% (95%CI 93, 97%) alive at 12 months, 85% (95%CI 80, 89%) at 36 months, and 78% (95%CI 71, 84%) at 60 months follow-up. Survival was adversely affected by age, with a 6% increase in the risk of death for every year of advancing age at baseline (HR = 1.06, 95%CI 1.03, 1.09, *p* < 0.001). Survival outcomes were better for women with low-grade, early-stage endometrial cancer (*p* < 0.001). LVSI and deep myometrial invasion were adverse prognostic factors (HR = 2.59, 95%CI 1.48, 4.50, *p* = 0.001 and HR = 2.43, 95%CI 1.39, 4.24, *p* = 0.002, respectively), as was a history of diabetes mellitus (HR = 2.84, 95%CI 1.58, 5.11, *p* ≤ 0.001). Compared to women whose primary treatment was surgery, those deemed unfit for hysterectomy (and had hormonal therapy) had a two-fold higher risk of death (HR = 2.42, 95%CI 1.20, 4.88, *p* = 0.013), mostly from causes unrelated to cancer, while those who received palliative radiotherapy had a 9-fold increased risk of death (HR = 9.25, 95%CI 2.19, 39.1, *p* = 0.002).

Women with a clinical history of hypothyroidism had better survival outcomes (HR = 0.34, 95%CI 0.11, 1.10), although this did not reach statistical significance in the unadjusted model (*p* = 0.07). After adjusting for age, BMI, socioeconomic deprivation quintile, type 2 diabetes mellitus, histological subtype, LVSI, depth of myometrial invasion, stage and grade of endometrial cancer, women with a history of hypothyroidism had a 78% reduced risk of death (adjusted HR = 0.22, 95%CI 0.06, 0.74, *p* = 0.02) compared to those without (Table 4).

**Figure 1:**
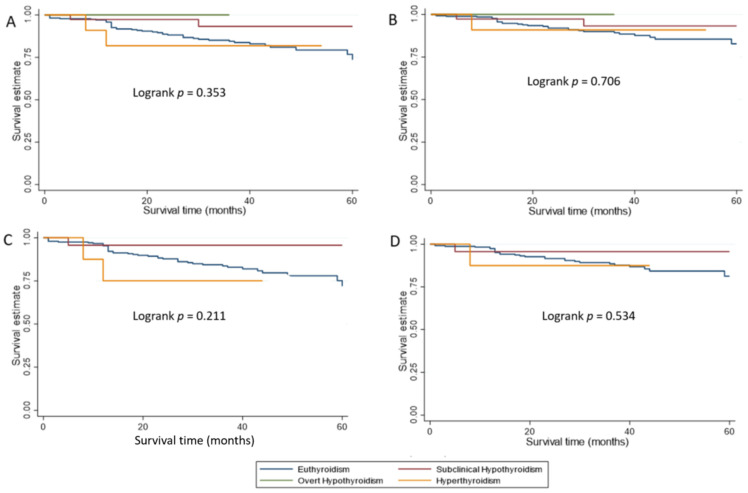
Kaplan–Meier survival curves showing survival estimates by biochemical thyroid status at endometrial cancer diagnosis. (**A**) Overall survival estimates for the whole study cohort. (**B**) Cancer-specific survival estimates for the whole cohort. (**C**) Overall survival estimates for the cohort with no history of hypothyroidism. (**D**) Cancer-specific survival estimates for the cohort with no history of hypothyroidism.

There was no statistically significant difference in overall survival by biochemical thyroid status at endometrial cancer diagnosis either the whole cohort or in those without a history of hypothyroid (Figure 1A,C).

Women with biochemical evidence of subclinical hypothyroidism had a 69% reduced risk of death (HR 0.31, 95%CI 0.09, 1.28) compared to euthyroid women while those with hyperthyroidism had a 21% increased risk (HR 1.21, 95%CI 0.29, 5.01), although these were not statistically significant (*p*-values 0.106 and 0.789, respectively). After adjustment for confounding factors, those with biochemical evidence of subclinical hypothyroidism had a 46% reduced risk of death (HR 0.54, 95%CI 0.12, 2.43) compared to euthyroid women while hyperthyroid women had a 67% increased risk (HR 1.67, 95%CI 0.37, 7.50,). The results remained statistically non-significant (*p*-values 0.424 and 0.503, respectively). We are unable to comment on the risk pattern for women with overt biochemical hypothyroidism due to the small numbers.

There was no evidence of an association between baseline TSH level and overall survival, either when considering TSH as a continuous variable (adjusted HR = 0.98, 95%CI 0.85, 1.26, *p* = 0.810 per unit increase) or at the clinically relevant cut-off of 4.5 mU/L (adjusted HR = 0.48, 95%CI 0.14, 1.65, *p* = 0.25). There was also no evidence of an association between baseline FT4, TT3 or FT4/T3 ratio and overall survival (adjusted HR = 0.99, 95%CI 0.89, 1.11, *p* = 0.872 adjusted HR = 1.02, 95%CI 0.40, 2.60, *p* = 0.973, and HR 1.01, 95%CI 0.89, 1.15, *p* = 0.835, respectively). We were unable to assess the impact of TPO antibodies on overall survival in the hypothyroid cohort due to the small number of events. However, for the whole cohort, there was no evidence of an association between baseline TPO antibody levels and overall survival (adjusted HR = 1.00, 95%CI 0.99, 1.01, *p* = 0.716).

### 3.4. Recurrence-Free and Cancer-Specific Survival Outcomes

Over the study period, 38 (11.7%) women relapsed with a median time to recurrence of 13 months (IQR 7, 21). The overall recurrence-free survival rate was 93% (95%CI 89%, 95%) at 12 months, 86% (95%CI 81%, 90%) at 36 months and 81% (95%CI 72%, 87%) at 60 months. Histological subtype, FIGO stage, grade, and history of type 2 diabetes mellitus were important predictors of disease recurrence (Table 5). Five-year recurrence-free survival rates were 94% and 78% in women with and without a clinical history of hypothyroidism, respectively. Women with a history of hypothyroidism had an 83% reduced risk of recurrence in multivariable analysis (adjusted HR = 0.17, 95%CI 0.04, 0.77, *p* = 0.02).

There was no evidence of an association between biochemical thyroid status at endometrial cancer diagnosis and disease recurrence. There was no evidence of an association between baseline TSH and risk of recurrence (adjusted HR = 0.57, 95%CI 0.16, 2.05, *p* = 0.39 at TSH > 4.5 mU/L). When analysed as continuous variables, there was no evidence of an association between baseline FT4, TT3, FT4/T3, or TPO antibodies and disease recurrence in adjusted or unadjusted analyses.

Women with a clinical history of hypothyroidism had a 79% reduced risk of cancer-specific mortality (adjusted HR = 0.21, 95%CI 0.05, 0.98, *p* = 0.04) compared to those without. There was no evidence of an association between cancer-specific survival and biochemical thyroid status at endometrial cancer diagnosis in either the whole cohort or in those without a history of hypothyroid (Figure 1B,D). Adjusted hazard ratios for women with biochemical evidence of subclinical hypothyroidism and hyperthyroidism were 0.68 (95%CI 0.14, 3.29, *p* = 0.627) and 1.59 (95% CI 0.19, 13.26, *p* = 0.666) respectively, in comparison to euthyroid women.

There was also no evidence of an association between serum thyroid hormone levels at baseline (TSH, FT4, TT3, FT4/T3, nor TPO antibody levels) and cancer-specific mortality.

## 4. Discussion

In this study, we explored the impact of clinical and biochemical thyroid dysfunction on the survival outcomes of a cohort of women treated for endometrial cancer in the North West of England. In total, 15.3% of women had a prior diagnosis of hypothyroidism, and a further 8.5% had biochemical evidence of subclinical hypothyroidism on baseline bloods. Women with a diagnosis of hypothyroidism had improved overall, endometrial cancer-specific, and recurrence-free survival compared to those without. After adjustment for known confounders, there was no statistically significant evidence of an association between survival outcomes and biochemical thyroid dysfunction at endometrial cancer diagnosis. However, we observed that women with evidence of subclinical hypothyroidism had improved survival compared to those who were euthyroid, and those with evidence of hyperthyroidism had worse survival. More research is now needed to confirm these findings, particularly given their potential therapeutic implications.

Our findings support much of the current literature regarding thyroid dysfunction and survival outcomes in cancer. A link was first suggested over a century ago [29], and since then, epidemiological and clinical trial data suggest that thyroid dysfunction not only influences the risk of certain solid cancers but also impacts survival after treatment for those cancers [5,22,30]. There is accumulating evidence from pre-clinical and animal models to support a role for thyroid hormones in cancer initiation, growth, and progression that can be reversed by their biochemical or surgical withdrawal [5,6,7]. In health, thyroid hormones influence metabolism through binding to the intranuclear thyroid hormone receptors (TR) α and β. This activates downstream signalling pathways and triggers the production of transcription factors that regulate metabolic function [7]. Thyroid hormones also bind to integrin αvβ3 on the cell surface to activate the phosphatidylinositol-3-kinase (PI3K-Akt) and the mammalian target of rapamycin (-mTOR) signalling pathways as well as the mitogen-activated protein kinase/extracellular signal-related kinase 1/2 (MAPK/ERK 1/2) pathways, with downstream effects on metabolism and cellular proliferation [31].

In cancer, changes in circulating levels of thyroid hormones due to thyroid disease and/or altered tumour expression of their receptors may impact tumour growth, invasion, and metastatic potential, and this has been reported in several cancer types, including glioma, myeloma, breast, lung and ovarian cancer [7]. Pique and colleagues applied a novel computational tool, receptLoss, to gene expression data from The CancerGenome Atlas (TCGA) and found an association between thyroid hormone receptor beta (THRB) expression loss and increased five-year survival in women with endometrial cancer [32]. Tumours with THRB expression loss were noted to be enriched with microsatellite instable molecular subtypes of endometrial cancer [32]. The thyroid hormone receptor beta, encoded by the THRB gene, plays a crucial role in normal development, growth, and metabolism. A gain of function mutation in the THRB gene leads to resistance to thyroid hormone syndrome, a condition characterised by elevated thyroid hormone levels, normal or mildly elevated TSH, and goitre [33]. An expression loss of THRB is therefore expected to result in reduced thyroid hormone levels. Thus, the finding by Pique and colleagues is consistent with our finding of an improved survival outcome in women with a diagnosis of hypothyroidism. Shinderman-Mama et al. (2016) demonstrated that integrin αvβ3 is overexpressed in ovarian cancer, and its activation stimulates tumour growth and migration, suggesting inhibition of this axis could provide a therapeutic strategy [34]. Crosstalk between thyroid hormone activation of integrin αvβ3 and oestrogen signalling pathways has been shown to promote cancer cell proliferation in oestrogen-driven tumours [35]. For instance, thyroid hormone activation of the MAPK/ERK 1/2 pathway in breast cancer cells phosphorylates oestrogen receptor-α (ERα), which in turn promotes cellular proliferation and tumour growth [36,37]. Crosstalk between thyroid hormone activation of integrin αvβ3 and ERα has also been shown to stimulate ovarian cancer growth [38]. Around 80% of endometrial cancers are endometrioid tumours caused by overstimulation of the PI3K-Akt-mTOR and MAPK/ERK1/2 signalling pathways by unopposed oestrogen [39]. Upregulation of integrin αvβ3 by endometrial cancer cells has been associated with aggressive tumour biology [40]. Integrin αvβ3 is expressed on both endometrioid and serous-type endometrial tumour cells, suggesting that signalling via this pathway is common across both type I and type II endometrial cancers [41,42]. The generally low levels of T4 in women with hypothyroidism would be expected to have less effect on integrin avβ3 expressed in both type I and II endometrial cancers, leading to suitable outcomes. We hypothesise that increased physiological levels of circulating thyroid hormone promote cell growth and invasiveness in both type I and type II endometrial cancers, while reduced concentrations, as seen in clinical hypothyroidism, have the opposite effect. Indeed, in our study, those with biochemical evidence of subclinical hypothyroidism had improved overall survival compared with those who were euthyroid (HR 0.31, 95%CI 0.09, 1.28), whereas those with hyperthyroidism had a worse survival (HR 1.21, 95%CI 0.29, 5.01), although these findings did not reach statistical significance. This may be explained by the relatively small number of patients in our cohort with biochemical thyroid dysfunction at endometrial cancer diagnosis, and therefore were underpowered to show an effect. The number of women with overt biochemical hypothyroidism at baseline was too small to comment on.

Clinically, those with a history of hypothyroidism who are adequately treated with thyroid hormone replacement are considered euthyroid, reflected by their serum TSH. In our cohort, 58% of women with a clinical history of hypothyroidism were biochemically euthyroid, and a further 27% were found to have subclinical hypothyroidism at endometrial cancer diagnosis. What is unexpected is that despite only 15% demonstrating overt biochemical thyroid dysfunction at the time of endometrial cancer diagnosis, women with a clinical history were observed to have significantly improved survival outcomes. Women with a diagnosis of hypothyroidism had a significantly higher T4:T3 ratio than those without (median 10.7 vs. 8.1, *p* = 0.001). These findings have been observed in a large retrospective study of 1692 patients with metastatic brain cancer, whereby those with a history of hypothyroidism had longer median survival than those who did not (31 months vs. 21 months, *p* = 0.0026) and was independently associated with survival in the multivariate analysis (HR = 0.76, 95%CI 0.63–0.91, *p* = 0.0034) [15]. Levothyroxine (T4) monotherapy is standard management for hypothyroidism, and once commenced, the dosage is titrated until serum TSH is within the normal range [43]. However, there is evidence to suggest that serum TSH may not reflect the thyroid status of peripheral tissues. T4 is converted to the bioactive T3 intracellularly in peripheral tissues by type 1 and type 2 iodothyronine deiodinases [44]. It has been shown that patients treated with levothyroxine may not achieve the required serum levels of T3, suggesting inadequate peripheral conversion leading to an increased T4:T3 ratio that, in a number of patients, never reaches the level required of those who are euthyroid despite a normal serum TSH, and this is observed more frequently in the female and aged populations [45]. This suggests that despite adequate thyroid hormone replacement and normal serum TSH, there may be persistent intracellular hypothyroid status in peripheral tissues and tumour tissues, with downstream consequences for cellular proliferation and growth, a hypothesis that might explain our findings.

Only one previous study investigated the association between thyroid function and survival after treatment for endometrial cancer [20]. Women with a pre-treatment TSH > 2.5 mU/L had a 2-fold increased risk of recurrence (HR = 2.1, 95%CI 0.9–4.8, *p* 0.057) and a nearly 3-fold increased disease-specific mortality (HR = 2.7, 95%CI 1.1–6.7, *p* 0.03) than women with TSH < 2.5 mU/L. While these findings directly contradict ours. There are some important differences between the studies that limit their comparability. First, the authors did not have information on clinical thyroid status for their study participants. Second, they base their analysis on a TSH cut-off that does not mirror that used in current clinical practice [27]. Third, they did not include T4 and T3 in their analyses, and therefore we do not know the impact of these hormones and biochemical thyroid status at baseline on survival. While 26/199 patients had TSH > 2.5 mU/L, we do not know how many had TSH > 4.5 mU/L, and the number is likely too low to allow robust conclusions. Fourth, they did not see the expected relationship between age, histological subtype, and endometrial cancer survival outcomes and failed to adjust for LVSI or myometrial invasion, key prognostic variables that could explain their results.

We found a 15% prevalence of clinical hypothyroidism in women newly diagnosed with endometrial cancer. Most were biochemically euthyroid, but 30% had elevated serum TSH levels, suggesting suboptimal thyroid replacement therapy or inappropriate reference ranges [46,47]. There were very few women with overt, untreated thyroid disease, but 16% of the study population had biochemical evidence of an under or over-active thyroid at baseline. This is a higher rate of thyroid dysfunction than expected, even when taking into account the female sex and age profile of the cohort [48]. In addition, two-thirds of our patients were obese, and nearly one-fifth had a diagnosis of type 2 diabetes mellitus. A high rate of obesity, insulin resistance, and metabolic dysfunction is well documented amongst endometrial cancer survivors [49,50] and contributes to their increased risk of cardiovascular disease [51,52,53]. Thus, optimising body weight, promoting healthy eating and exercise, and screening for and treating cardiovascular risk factors are all important considerations after treatment for endometrial cancer [54,55]. It is intriguing that a history of hypothyroidism is associated with better survival outcomes in this group. If confirmed in larger studies, it could provide a therapeutic strategy for poor prognosis endometrial cancer patients. In metastatic renal cell carcinoma, sunitinib-induced hypothyroidism is associated with improved progression-free survival [8,9,10], and thyroid dysfunction caused by immune checkpoint inhibition portends suitable outcomes in several cancer types [56,57]. Inhibition of the cell adhesion molecule integrin αvβ3 could provide a novel targeted treatment strategy for endometrial and other cancers. Of course, our findings remain an association only, and we cannot prove a causal relationship. Further larger pre-clinical and clinical studies are required to confirm our findings and to identify the underlying causal mechanisms to allow the development of potential treatment targets.

To our knowledge, this is the first study to explore the impact of clinical and biochemical thyroid dysfunction on endometrial cancer survival outcomes. Strengths of our work include the large, prospectively maintained database with detailed clinico-pathological data and survival outcomes over a 35-month median follow-up. Survival events were collected from clinic letters and cause of death verified from death certificates, and so were up to date and accurate; however, the small number of events observed in our cohort may be a limitation of our work. While a diagnosis of hypothyroidism correlated with survival outcomes, biochemical thyroid dysfunction at endometrial cancer diagnosis did not. This may be explained by the fact that women with a diagnosis of hypothyroidism were already on levothyroxine, which would have altered their thyroid function at study entry. The diagnosis of hypothyroidism was confirmed by GP records; however, we did not record the duration of symptoms, date of diagnosis, or dose of thyroid replacement therapy. While the classically applied endometrial cancer prognostic parameters such as stage, grade, histological subtype, lymphovascular space invasion, and depth of myometrial invasion all demonstrated the expected prognostic associations with endometrial cancer, the relatively low number of women with a diagnosis of hypothyroidism affects the reliability of our conclusions. We were only able to measure biochemical thyroid function at baseline, which provides a snapshot of thyroid status at the time of endometrial cancer diagnosis, but does not take into account biological variations or changes in thyroid replacement therapy over time. We did not prescribe levothyroxine for women with subclinical hypothyroidism on their baseline bloods, and neither did we record clinical thyroid dysfunction diagnosed during follow-up. The lack of smoking status data is a limitation since smoking is associated with lower serum TSH and TPOAb levels [58]. This may be relevant if smoking has an adverse effect on endometrial cancer survival outcomes. The single centre nature of our study is a weakness since we cannot necessarily extrapolate our findings to other cancer centres in the U.K. or elsewhere, where case mix and prevalence of clinical and biochemical thyroid dysfunction in women newly diagnosed with endometrial cancer may differ.

## 5. Conclusions

In summary, we report an association between a clinical history of hypothyroidism and endometrial cancer survival outcomes. Large, well-designed longitudinal studies are now needed to confirm this finding and elucidate the mechanisms underpinning it.

## Figures and Tables

**Table 1:** Socio-demographic and clinical distribution of the study population (*n* = 333).

Variable	Number (% of Total)
Age at diagnosis (years)	Median (IQR)—66 (50, 82)
<50 years	42 (12.6%)
50–70 years	178 (53.5%)
>70 years	113 (33.9%)
Body Mass Index (kg/m^2^)	Median (IQR)—33 (19, 47)
Underweight	3 (0.9%)
Normal weight	45 (13.5%)
Overweight	70 (21.0%)
Obese	215 (64.6%)
Social deprivation quintile (*n* = 331)	
1 (Most deprived)	125 (37.8%)
2	69 (20.9%)
3	41 (12.4%)
4	52 (15.7%)
5 (Least deprived)	44 (13.3%)
History of hypothyroidism	
Yes	51 (15.3%)
No	282 (84.7%)
History of diabetes mellitus	
Yes	59 (17.7%)
No	274 (82.3%)
Histological subtype	
Endometrioid	268 (80.5%)
Serous	24 (7.2%)
Carcinosarcoma	21 (6.3%)
Clear cell	12 (3.6%)
Other	8 (2.4%)
FIGO (2009) Stage	
I	256 (76.9%)
II	29 (8.7%)
III	43 (12.9%)
IV	5 (1.5%)
Grade	
1	164 (49.3%)
2	75 (22.5%)
3	94 (28.2%)
LVSI (*n* = 332)	
No	239 (72%)
Yes	93 (28%)
Depth of myometrial invasion (*n* = 332)	
<50%	217 (65.4%)
≥50%	115 (34.6%)
Primary treatment	
Surgery	268 (80.5%)
Hormonal (Fertility sparing)	25 (7.5%)
Hormonal (Not fit for surgery)	37 (11.1%)
Radiotherapy	3 (0.9%)
Adjuvant treatment	
Yes	132 (39.6%)
No	201 (60.4%)
Recurrence (*n* = 326)	
Yes	38 (11.7%)
No	288 (88.3%)
Overall survival status at end of follow-up	
Alive	283 (85.0%)
Cancer-specific mortality	33 (9.9%)
Non-cancer related mortality	17 (5.1%)

LVSI—lymphovascular space invasion. IQR—interquartile range.

**Table 2:** Clinical and biochemical thyroid function at baseline.

Variable	Women with No History of Hypothyroidism *n* = 282 (84.7%)	Women with History of Hypothyroidism *n* = 51 (15.3%)	All Women *n* = 333 (100%)
Biochemical thyroid patterns, *n* (%)			
Euthyroid (Normal TSH, normal FT4)	240 (88.2%)	28 (58.3%)	268 (83.8%)
Hypothyroid	23 (8.5%)	13 (27.1%)	36 (11.3%)
Subclinical (High TSH, normal FT4)			
Overt (High TSH, low FT4)	-	2 (4.2%)	2 (0.6%)
Secondary (Normal TSH, low FT4)	1 (0.4%)	1 (2.1%)	2 (0.6%)
Hyperthyroid	7 (2.6%)	2 (4.2%)	9 (2.8%)
Subclinical (Low TSH, normal FT4)			
Overt (Low TSH, high FT4)	1 (0.4%)	1 (2.1%)	2 (0.6%)
High FT4, normal TSH	-	1 (2.1%)	1 (0.3%)
Total	272 (100%)	48 (100%)	320 (100%)
Biochemical tests, *n* (%)			
TSH (mU/L) (*n* = 329)	median (IQR) 2.1 (0.12, 9.1) *n* = 279	median (IQR) 2.2 (0.003, 40.0) *n* = 50	median (IQR) 2.1 (0.003, 40.0)
<0.4	8 (2.9%)	3 (6.0%)	11 (3.30%)
0.4–4.5	247 (88.5%)	32 (64.0%)	279 (84.8%)
>4.5	24 (8.6%)	15 (30.0%)	39 (11.9%)
FT4 (pmoL/L) (*n* = 323)	median (IQR) 14.0 (7.3, 27.2) *n* = 274	median (IQR) 15.7 (7.7, 31.0) *n* = 49	median (IQR) 14.2 (7.3, 31.0)
<9.0	1 (0.4%)	3 (6.1%)	4 (1.2%)
9–25	272 (99.3%)	44 (89.8%)	316 (97.8%)
>25	1 (0.4%)	2 (4.1%)	3 (0.9%)
TT3 (nmoL/L) (*n* = 331)	mean (SD) 1.73 (0.33) *n* = 280	mean (SD) 1.53 (0.37) *n* = 51	mean (SD) 1.70 (0.34)
<1.2	8 (2.9%)	9 (17.7%)	17 (5.1%)
1.2–2.6	267 (95.4%)	41 (80.45)	308 (93.1%)
>2.6	5 (1.8%)	1 (2.0%)	6 (1.8%)
TPOAb (IU) (*n* = 321)			
<60 (negative)	252 (92.6%)	34 (69.4%)	286 (89.1%)
≥60 (positive)	20 (7.4%)	15 (30.6%)	35 (10.9%)
TSH prognostic categories (*n* = 329)			
≤4.5 mU/L	255 (91.4%)	35 (70.0%)	290 (88.1%)
>4.5 mU/L	24 (8.6%)	15 (30.0%)	39 (11.9%)
Total	282 (100%)	51 (100%)	333 (100%)

Footnote: Complete test results enabling characterisation of the thyroid biochemical patterns (euthyroid, hypothyroid, and hyperthyroid status) as per the British Thyroid Association guidelines were available for 320 women. *n*—number. IQR—interquartile rage. SD—standard deviation. TSH—thyroid-stimulating hormone. FT4—free T4. TT3—total T3. TPOAb—thyroid peroxidase antibodies.

**Table 3:** One-, three-, and five-year overall survival rates and unadjusted hazard ratios with 95% confidence intervals by demographic and clinical predictor factors.

Variable	One Year Survival % (95% CI)	Three-Year Survival % (95% CI)	Five-Year Survival % (95% CI)	Hazard Ratio (95% CI)	*p*-Value
Age (years)					
<50	100	95 (72, 99)	95 (72, 99)	1.00	
50–70	96 (92, 98)	87 (81, 92)	80 (70, 88)	5.49 (0.74, 40.6)	0.095
>70	93 (87, 97)	77 (67, 84)	69 (54, 81)	10.57 (1.43, 78.0)	**0.021**
BMI categories					
Normal weight	100	95 (82, 98)	85 (49, 96)	1.00	
Overweight	96 (87, 99)	76 (63, 85)	71 (56, 81)	3.08 (1.03, 9.22)	**0.045**
Obese	95 (91, 97)	85 (79, 90)	80 (72, 87)	1.84 (0.65, 5.24)	0.254
Social deprivation quintile					
Quintile 1 (Most deprived)	96 (90, 98)	85 (79, 90)	80 (72, 87)	1.00	
Quintile 2	93 (83, 97)	86 (76, 92)	84 (74, 90)	1.40 (0.64, 3.04)	0.401
Quintile 3	100	82 (69, 90)	82 (69, 90)	1.08 (0.42, 2.78)	0.878
Quintile 4	96 (85, 99)	83 (66, 92)	83 (66, 92)	1.06 (0.43, 2.61)	0.895
Quintile 5 (Least deprived)	95 (81, 99)	89 (75, 95)	77 (53, 90)	2.19 (1.00, 4.76)	**0.049**
History of diabetes mellitus					
No	96 (93, 98)	88 (83, 92)	83 (75, 89)	1.00	
Yes	93 (82, 97)	68 (52, 80)	55 (34, 72)	2.84 (1.58, 5.11)	**<0.001**
History of hypothyroidism					
No	95 (91, 97)	83 (79, 88)	76 (67, 83)	1.00	
Yes	100	93 (78, 98)	93 (78, 98)	0.34 (0.11, 1.10)	0.072
Baseline TSH (mU/L)					
≤4.5	95 (92, 97)	84 (79, 88)	77 (68, 84)	1.00	
>4.5	97 (82, 100)	89 (69, 96)	89 (69, 96)	0.49 (0.15, 1.59)	0.237
Histological subtype					
Endometrioid	97 (94, 98)	89 (84, 92)	85 (78, 90)	1.00	
Non-endometrioid	91 (80, 96)	68 (54, 79)	46 (17, 70)	3.37 (1.90, 5.97)	**<0.001**
FIGO (2009) Stage					
Early Stage (I/II)	98 (95, 99)	88 (83, 92)	82 (74, 88)	1.00	
Late stage (III/IV)	83 (69, 91)	66 (49, 78)	57 (39, 72)	3.45 (1.92, 6.20)	**<0.001**
Grade					
1	97 (93, 99)	91 (84, 95)	86 (76, 92)	1.00	
2	97 (89, 99)	89 (78, 95)	87 (75, 93)	1.20 (0.52, 2.80)	0.666
3	92 (85, 96)	72 (60, 80)	55 (33, 72)	3.72 (1.95, 7.11)	**<0.001**
LVSI					
No	97 (94, 99)	89 (84, 93)	83 (73, 89)	1.00	
Yes	92 (84, 96)	73 (62, 81)	68 (55, 79)	2.59 (1.48, 4.50)	**0.001**
Depth of myometrial invasion					
≤50%	97 (94, 99)	90 (84, 93)	83 (73, 90)	1.00	
>50%	93 (86, 96)	75 (64, 82)	70 (58, 79)	2.43 (1.39, 4.24)	**0.002**

BMI—body mass index. CI—confidence interval. TSH—thyroid-stimulating hormone. LVSI—lymphovascular space invasion. Bold values denote statistical significance at the *p* < 0.05 level.

**Table 4:** Adjusted Cox regression analysis of TSH prognostic groupings and survival from endometrial cancer.

Thyroid Marker	Hazard Ratio (95% CI)	*p*-Value
Overall Survival		
History of Hypothyroidism		
No	1.00	
Yes	0.22 (0.06, 0.74)	**0.02**
Baseline TSH (mU/L)		
≤4.5	1.00	
>4.5	0.48 (0.14, 1.65)	0.25
Recurrence-free Survival		
History of Hypothyroidism		
No	1.00	
Yes	0.17 (0.04, 0.77)	**0.02**
Baseline TSH (mU/L)		
≤4.5	1.00	
>4.5	0.57 (0.16, 2.05)	0.39
Cancer-Specific Survival		
History of Hypothyroidism		
No	1.00	
Yes	0.21 (0.05, 0.98)	**0.04**
Baseline TSH (mU/L)		
≤4.5	1.00	
>4.5	0.38 (0.08, 1.82)	0.23

Adjusted for age, body mass index, social class, history of diabetes, FIGO stage, grade and histological subtype, lymphovascular space invasion, depth of myometrial invasion. CI—confidence interval. TSH—thyroid-stimulating hormone. Bold values denote statistical significance at the *p* < 0.05 level.

**Table 5:** One, three and five-year recurrence-free survival rates and unadjusted recurrence hazard ratios and 95% confidence intervals by TSH groupings, demographic and clinical predictor factors.

Variable	One Year Survival % (95% CI)	Three-Year Survival % (95% CI)	Five-Year Survival % (95% CI)	Hazard Ratio (95% CI)	*p*-Value
Age					
<50	100	97 (82, 99)	97 (82, 99)	1.00	
50–70	91 (85, 95)	84 (76, 89)	79 (69, 86)	6.32 (0.86, 46.8)	0.071
>70	94 (87, 98)	85 (74, 92)	74 (47, 89)	6.03 (0.78, 46.4)	0.084
BMI categories					
Normal weight	100	92 (76, 97)	81 (50, 94)	1.00	
Overweight	87 (75, 94)	81 (65, 90)	71 (44, 86)	2.15 (0.67, 6.85)	0.166
Obese	94 (90, 97)	87 (80, 91)	84 (75, 90)	1.34 (0.46, 3.89)	0.701
Social deprivation quintile					
Quintile 1 (Most deprived)	94 (87, 97)	84 (75, 90)	84 (75, 90)	1.00	
Quintile 2	94 (84, 98)	84 (70, 92)	84 (70, 92)	1.03 (0.45, 2.44)	0.944
Quintile 3	91 (75, 97)	83 (63, 93)	83 (63, 93)	1.01 (0.37, 2.78)	0.986
Quintile 4	100	95 (72, 99)	88 (57, 97)	0.29 (0.07, 1.25)	0.097
Quintile 5 (Least deprived)	86 (69, 94)	86 (69, 94)	56 (17, 83)	1.50 (0.07, 1.25)	0.380
History of diabetes mellitus					
No	94 (90, 97)	89 (84, 93)	82 (72, 89)	1.00	
Yes	87 (74, 94)	67 (47, 81)	67 (47, 81)	2.49 (1.26, 4.94)	**0.009**
History of hypothyroidism					
No	93 (89, 95)	85 (79, 89)	78 (67, 85)	1.00	
Yes	98 (84, 100)	94 (78, 99)	94 (78, 99)	0.28 (0.07, 1.16)	0.078
Baseline TSH (mU/L)					
≤4.5	93 (89, 96)	85 (80, 90)	79 (69, 86)	1.00	
>4.5	94 (79, 99)	88 (64, 96)	88 (64, 96)	0.66 (0.20, 2.16)	0.495
Histological subtype					
Endometrioid	96 (92, 97)	91 (85, 94)	89 (82, 93)	1.00	
Non-endometrioid	83 (71, 90)	67 (49, 79)	33 (6.4, 64)	4.56 (2.39, 8.69)	**<0.001**
FIGO (2009) Stage					
Early Stage (I/II)	97 (93, 98)	90 (85, 94)	84 (74, 91)	1.00	
Late stage (III/IV)	71 (55, 83)	59 (41, 73)	59 (41, 73)	5.55 (2.89, 10.7)	**<0.001**
Grade					
1	99 (95, 100)	96 (88, 99)	93 (78, 98)	1.00	
2	97 (89, 99)	88 (76, 94)	88 (76, 94)	3.22 (0.94, 11.0)	0.062
3	80 (70, 88)	67 (54, 77)	49 (26, 69)	13.64 (4.75, 39.2)	**<0.001**
LVSI					
No	97 (93, 98)	93 (88, 96)	88 (76, 94)	1.00	
Yes	84 (75, 91)	67 (54, 77)	62 (46, 74)	5.54 (2.83, 10.84)	**<0.001**
Depth of myometrial invasion					
≤50%	97 (93, 99)	92 (86, 96)	86 (74, 93)	1.00	
>50%	86 (77, 91)	72 (60, 81)	68 (53, 79)	3.82 (1.98, 7.40)	**<0.001**

BMI—body mass index. CI—confidence interval. TSH—thyroid-stimulating hormone. LVSI—lymphovascular space invasion. Bold values denote statistical significance at the *p* < 0.05 level.

## Data Availability

Anonymised data are available upon reasonable request to the corresponding author.
